# Effects of letrozole supplementation on growth performance, blood indexes, ruminal fermentation parameters, and microbiome composition of *hu* lambs

**DOI:** 10.3389/fmicb.2026.1734219

**Published:** 2026-02-26

**Authors:** Lukuan Yang, Tingting Li, Renping Liu, Yaqian Zhang, Munire Ainiwaer, Shanshan Wang, Zhiqiang Liu, Kailun Yang, Caidie Wang

**Affiliations:** 1 Xinjiang Herbivore Nutrition Laboratory for Meat and Milk, College of Animal Science and Technology, Xinjiang Agricultural University, Urumqi, Xinjiang, China; 2 Xinjiang Shangpin Meiyang Technology Co., Ltd., Changji, Xinjiang, China

**Keywords:** growth performance, lamb, letrozole, plasma hormone, rumen fermentation

## Abstract

This study aimed to explore the effects of dietary letrozole (LE) supplementation on growth performance, rumen microbiota, fermentation profiles, and blood metabolites in *Hu* lambs, providing insights into its potential for enhancing animal production. Twenty-eight male *Hu* lambs (20.21 kg ± 0.56 kg, 70 days old) were randomly assigned to four groups, with seven replicates per group: a control group (CON), and three test groups (T1, T2, T3). Lambs in the CON group were fed a basal diet, while T1, T2, and T3 groups received 0.05, 0.1, and 0.2 mg/kg BW of LE, respectively, in addition to the basal diet. The experiment lasted for 46 days. The findings were as follows: (1) There were no significant differences among groups in Initial Body Weight (IBW), Final Body Weight (FBW), Average Daily Feed Intake (ADFI), Average Daily Gain (ADG), and feed conversion ratio throughout the entire trial (*P* > 0.05). (2) Compared with the CON group, plasma testosterone (T) concentrations in Groups T2 and T3 were significantly elevated at 0 h post-supplementation (*P* < 0.05). Compared with the control group, nitric oxide (NO) levels in Groups T1 and T2 were significantly reduced 4 h after LE supplementation (*P* < 0.05). (3)Nitric oxide (NO) levels in experimental groups exhibited a secondary change 4 h after supplementation (*P* < 0.05). There were no significant differences in plasma Total Antioxidant Capacity (T-AOC), Catalase (CAT), Superoxide Dismutase (SOD), Glutathione Peroxidase (GSH-Px), or Malondialdehyde (MDA) levels between 0 h pre-supplementation and 4 h post-supplementation across all experimental groups (*P* > 0.05). At 0 h before and 4 h after supplementation, Total Protein (TP), Albumin (ALB), and Globulin (GLB) levels in all experimental groups showed no significant differences compared to the CON group (*P* > 0.05). (4) Ammonia nitrogen (NH_3_-N) levels were extremely significantly higher in all test groups compared to the CON group (*P* < 0.05). Propionic acid and isovaleric acid concentrations in Group T3 were significantly higher than in the CON group (*P* < 0.01), while the ethyl-to-propyl ratio was significantly lower (*P* < 0.01). (5) At the phylum level, LE-treated groups showed a higher relative abundance of *Firmicutes* than the CON group (21.04%), with increases proportional to the LE dose: Group T3 (37.88%), Group T2 (32.74%), and Group T1 (30.66%). At the family level, the relative abundance of *Prevotellaceae* was significantly lower in all test groups compared to the CON group (*P* < 0.05), while *Lachnospiraceae* abundance was significantly higher in the test groups (*P* < 0.01). Under the experimental conditions, supplemental feeding of LE did not significantly affect the overall growth performance of lambs. but it did increase plasma testosterone concentration, elevated the relative abundance of Firmicutes in the rumen, reduced the relative abundance of Bacteroidetes, and altered the rumen fermentation pattern. This shift occurred by decreasing the acetate-to-propionate ratio, increasing isovaleric acid concentration, and promoting a propionic acid fermentation pattern, thereby improving feed utilization. Among all groups, the optimal supplemental feeding rate was determined to be 0.2 mg/kg BW.

## 1 Introduction

Reproductive hormones, as a key component of the endocrine system, are critical for physiological processes such as skeletal development, muscle growth, and fat metabolism in young livestock (Yuting et al., 2011). Androgens have been shown to accelerate protein synthesis, promote muscle growth, and regulate glucose and lipid metabolism, thereby improving growth performance (Schiffer et al., 2017). In contrast, estrogen plays a pivotal role in skeletal maturation, epiphyseal closure, and the cessation of linear growth (Li, 2024). Letrozole (LE), a third-generation aromatase inhibitor, works by specifically binding to the aromatase enzyme, thereby suppressing the conversion of androgens to estrogens and increasing androgen levels within the organism. A study by Rezaei et al. (2020) demonstrated that administering LE at a dosage of 0.25 mg/kg significantly increased serum testosterone (T) levels in goats, which correlated with enhanced average daily gain (ADG) and carcass weight. Furthermore, the microbiota has been shown to play significant roles in the reproductive endocrine system by interacting with hormones such as estrogen, androgen, and insulin, while also influencing the ruminal microbial structure and metabolic function (Rosales-Nieto et al., 2021; Tian, 2022). In ruminants, steroid hormones reach the rumen either through transmural diffusion across the ruminal wall or via saliva (Han, 2010), and these hormones subsequently modulate ruminal metabolic activity (Li et al., 2022).

Currently, research on the mechanism by which LE regulates growth performance in ruminants has primarily focused on endocrine pathways, while its effects on rumen microbial communities and metabolic products remain unclear. Therefore, this study used *Hu* sheep as the experimental subjects. By supplementing diets with varying levels of LE, we investigated its effects on growth performance, serum hormone levels, rumen fermentation parameters, rumen microbial community structure, and metabolomic characteristics. The aim was to elucidate the rumen microbial mechanisms through which LE regulates growth in Hu sheep, thereby providing a theoretical basis for the rational application of LE in ruminant production.

## 2 Materials and methods

### 2.1 Design and management of experiment

The trial was conducted at Xinjiang Shangpin Meiyang Technology Co., Ltd. (87.136291°E, 44.359568°N) from September to October 2023, lasting for 46 days. Twenty-eight 70-day-old male *Hu* lambs, with an average body weight of 20.21 ± 0.56 kg and in good health, were randomly assigned to four groups (*n* = 7): CON Group (basal diet), Group T1 (basal diet + 0.05 mg/kg BW^–1^ LE), Group T2 (basal diet + 0.1 mg/kg BW LE), and Group T3 (basal diet + 0.2 mg/kg BW LE). LE was dissolved in 1% carboxymethyl cellulose (CMC) and administered daily at 09:00 a.m. The basal diet was provided via a TMR feeding truck at 09:00 and 17:30 daily. The proportion and detailed composition of TMR are shown in Table 1. All lambs had a libitum access to both feed and water.

**TABLE 1:** Composition and nutritional level of basic diet (%).

Items		Contents	
Ingredient		Nutrient level	
Corn	30.00	ME/(MJ⋅kg^–1^)^➁^	9.45
Whole plant corn silage	24.00	DM	94.15
Alfalfa	20.00	CP	12.25
Corn germ meal	16.00	EE	4.26
Corn bran	8.00	Ash	8.66
Premix➀	2.00	NDF	26.67
Total	100.00	ADF	18.76
		Ca	0.54
		P	0.35

➀ Premix provides the following per kg: Vitamin A 12,100 IU; Vitamin D2 1,150 IU; Vitamin E 130 IU; Cu 18 mg; Zn 65 mg; Mn 50 mg; Fe 65 mg; I 0.8 mg; Co 0.7 mg; Se 0.8 mg.

➁ Metabolizable energy was a calculated value, whereas nutrient levels were analytically determined values.

### 2.2 Sample collection

#### 2.2.1 Collection of growth performance indicators

The lambs’ body weight was measured after fasting on days 0, 15, 30, and 45, and *AverageDailyGain*(ADG) was calculated. From days 31 to 37, daily feed intake and leftovers were recorded to determine the Average Daily Feed Intake (ADFI) and feed conversion ratio (FCR) for each group.


ADG=(Final⁢Weight-Initial⁢Weight)÷Trial⁢Days

ADFI=Feed⁢Intake÷(Trial⁢Duration×Number⁢of⁢Sheep)

FCR=Daily⁢Feed⁢Intake÷Daily⁢Weight⁢Gain

#### 2.2.2 Collection of blood biochemical parameters

On the 25th day of the experiment, 5 healthy lambs with similar body weight were selected from each group. Blood samples were collected from the jugular vein using heparin sodium anticoagulant tubes at 0 h before and 4 h after supplementary feeding. The samples were centrifuged at 3,500 rpm for 15 min, and the plasma was transferred into 1.5 mL cryovials and stored at -20°C for subsequent analysis.

#### 2.2.3 Collection of ruminal fluid parameters

On day 46, five healthy lambs of similar body weight were selected from each group. Approximately 2 h post-supplementation, around 100 mL of ruminal fluid was collected from each lamb using a vacuum extraction device. To avoid contamination from saliva, the first 50 mL was discarded. The pH of the remaining ruminal fluid was immediately measured using a portable pH meter, and the fluid was then filtered through four layers of medical gauze, aliquoted, and stored at -80°C.

### 2.3 Sample analysis

#### 2.3.1 Plasma biochemical parameter assay

The plasma levels of T, E_2_, GH, and insulin were measured using the enzyme-linked immunosorbent assay (ELISA). The levels of T-AOC, CAT, SOD, GSH-Px, MDA, and NO concentration were determined using colorimetric methods. All these tests were commissioned to Beijing Huaying Biotechnology Co., Ltd. The Hua Wei Delong DR-200BS microplate reader was used (the instrument was purchased from Wuxi Hua Wei Delong Instrument Co., Ltd.). The analysis of plasma total protein (TP), albumin (ALB), and globulin (GLB) was outsourced to the Third People’s Hospital of Xinjiang.

#### 2.3.2 Rumen fluid parameter analysis

##### 2.3.2.1 NH_3_-H quantification

The NH_3_-N concentration was determined using the phenol-hypochlorite method (indophenol blue method), as detailed in Jin (2024). The analysis was performed using a TECAN Infinite M200 full-wavelength microplate reader (purchased from Tecan Group, Switzerland).

##### 2.3.2.2 VFA concentration assay

Volatile fatty acid (VFA) concentrations were determined according to the method described by Xu (2013) using a Shimadzu GC-2010 gas chromatograph, with 4-methylpentanoic acid serving as the internal standard.

##### 2.3.2.3 16 S rRNA gene amplicon sequencing of rumen bacteria

The 16S rRNA sequencing of rumen was commissioned to Beijing Novogene Biotech Co., Ltd. The V3-V4 hypervariable region of the bacterial 16S rRNA gene was amplified by PCR using universal bacterial primers (314F: 5’-CCTAYGGGRBGCASCAG-3’ and 806R: 5’-GGACTACNNGGGTATCTAAT-3’). Polymerase Chain Reaction (PCR) products were electrophoresed on a 2% agarose gel, purified with magnetic beads, and the target bands were extracted for sequencing library construction. Genome sequencing was performed on the Illumina Novaseq platform, and the resulting data were analyzed using QIIME2 software.

### 2.4 Data statistics and analysis

Data organization was initially performed in Microsoft Excel 2021. Statistical analyses were conducted separately: zootechnical and biochemical data were analyzed using one-way ANOVA followed by Duncan’s *post-hoc* test in SPSS 25.0, with significance levels set at *P* < 0.05 and *P* < 0.01 (Krzywinski and Altman, 2014). Microbial data, including alpha diversity indices and relative taxonomic abundances at the phylum, family, and genus levels, were analyzed via the Kruskal-Wallis test on the NovoMagic platform, which was also used for data visualization (Kruskal and Wallis, 2012).

## 3 Results

### 3.1 Sequencing quality control

**FIGURE 1:**
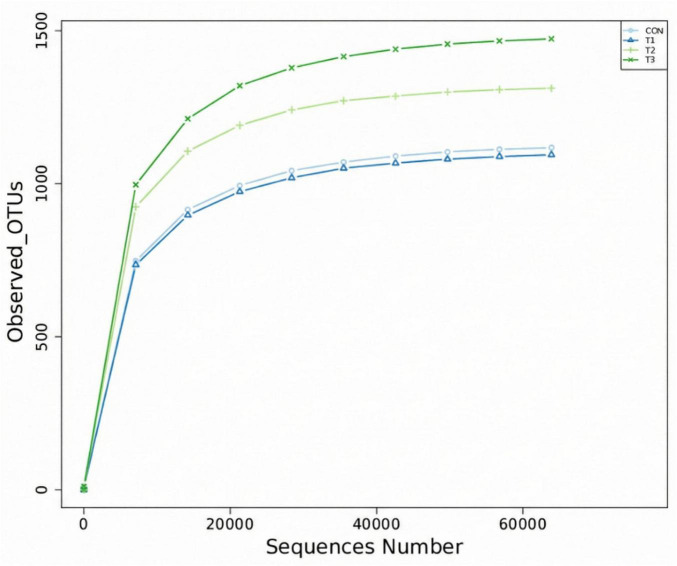
Rarefaction curve of samples.

The dilution curve construction is based on the relationship between the progressively increasing sequencing data volume and the corresponding α-diversity index values, as shown in Figure 1. As the sequencing data volume increases, the dilution curves for each sample group gradually plateau, indicating that the sequencing depth is sufficient to capture the major taxa within the microbial communities. This provides a comprehensive reflection of the microbial diversity and enables further analysis.

### 3.2 Microbial species composition

**FIGURE 2:**
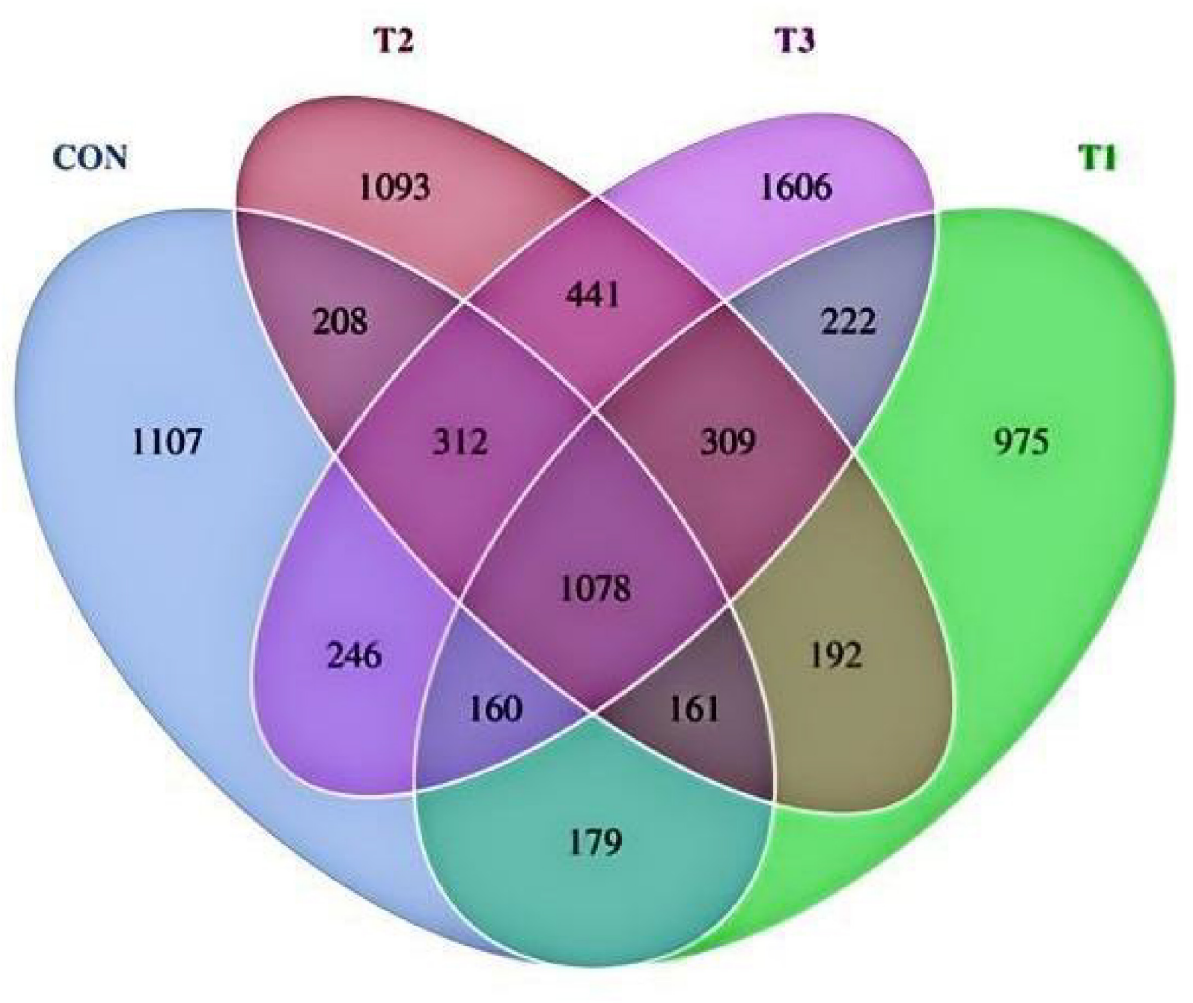
Effects of LE supplementation on rumen OTUs.

A total of 8,289 operational taxonomic units (OTUs) were identified across all samples in this study (Figure 2). Of these, 1,078 OTUs (13.00% of the total) were shared across all four groups. The numbers of unique OTUs in the CON, T1, T2, and T3 groups were 1,107 (13.36%), 975 (11.76%), 1,093 (13.19%), and 1,606 (19.38%), respectively.

### 3.3 Alpha diversity analysis

The Chao1 index in T3 was significantly higher than that in the CON and T1 groups (*P* < 0.05). No significant differences were observed in the Observed_otus, Shannon, or Simpson indices among the groups (*P* > 0.05). These results indicate that LE supplementation increased the richness of the rumen microbial community. Furthermore, the Goods coverage of all samples exceeded 99%, confirming that the sequencing depth was adequate to capture the majority of OTUs and reliably represent the microbial composition of the rumen fluid (Table 2).

**TABLE 2:** Effects of LE supplementation on alpha diversity index of rumen microbial community (*n* = 5).

Items	Groups				*P*-value
	CON	T1	T2	T3	
Chao1	1130.09^b^± 238.27	1126.08^b^± 169.91	1319.83^ab^± 109.65	1481.62^a^± 192.75	0.02
Goods_coverage	99.94 ± 0.02	99.94 ± 0.01	99.95 ± 0.01	99.93 ± 0.02	0.51
Observed_otus	1118.00 ± 239.09	1116.60 ± 168.86	1311.80 ± 107.38	1473.40 ± 191.61	0.01
Shannon	7.12 ± 0.74	7.52 ± 0.54	8.19 ± 0.52	8.28 ± 0.59	0.02
Simpson	0.96 ± 0.02	0.98 ± 0.01	0.98 ± 0.02	0.99 ± 0.01	0.12

Within a row, data points with no superscript letters or the same letter indicate no significant difference (*P* > 0.05). Different lowercase letters indicate a significant difference (*P* < 0.05), and different uppercase letters indicate a highly significant difference (*P* < 0.01).

### 3.4 PLS-DA analysis

**FIGURE 3:**
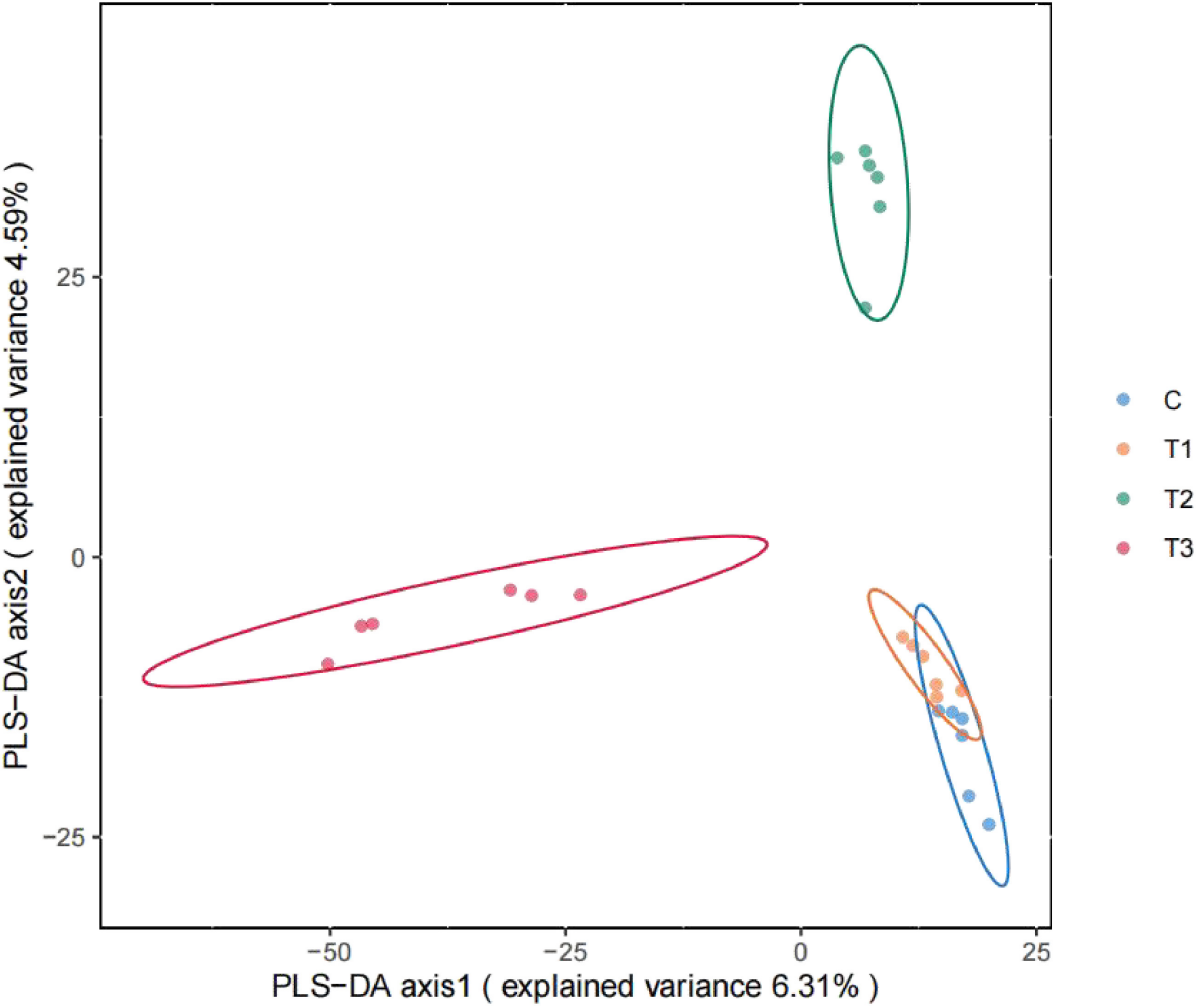
PLS-DA score plot.

The PLS-DA score plot, a tool commonly used to visually assess a model’s classification performance. The greater the separation between the two sample groups within the plot, the more pronounced the classification effect. The first principal component accounts for 6.31% of the variance, while the second principal component explains 4.59%. The CON group and Group T1 exhibit less distinct separation, which may be attributed to their smaller node sizes (Figure 3).

### 3.5 The effect of LE on rumen fermentation parameters in *hu* lambs

There is no significant differences in ruminal pH were observed among the groups (*P* > 0.05). However, the NH_3_-N concentration was significantly higher in the treatment groups compared to the CON group (*P* < 0.05). Specifically, groups T2 and T3 exhibited a highly significant increase in NH_3_-N concentration compared to group T1 (*P* < 0.01), with a linear increase in NH_3_-N levels corresponding to the escalating doses of LE. The concentrations of propionate and isovalerate in group T3 were significantly higher than those in the CON group and group T1 (*P* < 0.01), no significant difference compared with Group T2 (*P* > 0.05). Additionally, the isovalerate concentration demonstrated both linear and quadratic effects as the dose of LE supplementation increased. The acetate-to-propionate ratio was significantly lower in group T3 compared to the CON group (*P* < 0.01), with a linear decrease in Acetate-to-Propionate Ratio observed as LE supplementation increased. As shown in Table 3.

**TABLE 3:** The effect of feeding LE on rumen fermentation parameters of *Hu* lambs (*n* = 5).

Items	Groups				*SEM*	*P*-value		
	CON	T1	T2	T3		Anova	Linear	Quadratic
pH	6.72	6.80	6.74	6.85	0.03	0.28	0.15	0.85
NH3-N /(mg/dL)	11.70^Bc^	16.60^Bb^	24.00^Aa^	23.25^Aa^	1.37	< 0.01	< 0.01	0.10
Acetate/(mmol/L)	48.66	52.04	53.87	57.21	2.25	0.63	0.21	1.00
Propionate/(mmol/L)	14.45^Bb^	14.55^Bb^	18.27^ABab^	22.83^Aa^	1.10	< 0.01	< 0.01	0.21
Butyrate/(mmol/L)	10.93	10.07	10.22	10.59	0.43	0.91	0.84	0.51
Isobutyrate/(mmol/L)	1.28	1.35	1.28	1.67	0.07	0.19	0.10	0.28
Valerate/(mmol/L)	1.04	0.95	1.15	1.19	0.04	0.16	0.09	0.39
Isovalerate/(mmol/L)	1.51^Bb^	1.42^Bb^	1.74^ABb^	2.35^Aa^	0.11	< 0.01	< 0.01	0.03
Total VFAs)/(mmol/L)	77.87	80.38	86.54	95.85	3.47	0.28	0.06	0.62
Acetate-to-Propionate Ratio	3.41^ABa^	3.53^Aa^	2.94^ABab^	2.59^Bb^	0.12	0.01	< 0.01	0.24

Different lowercase letters on the same data point indicate significant differences (*P* < 0.05), different uppercase letters indicate highly significant differences (*P* < 0.01), and identical letters or no letters indicate no significant differences (*P* > 0.05).

### 3.6 The effect of LE on the rumen microbial flora of *hu* lambs

#### 3.6.1 Effect of LE supplementation on rumen microflora structure (phylum level) of *hu* lambs

**FIGURE 4:**
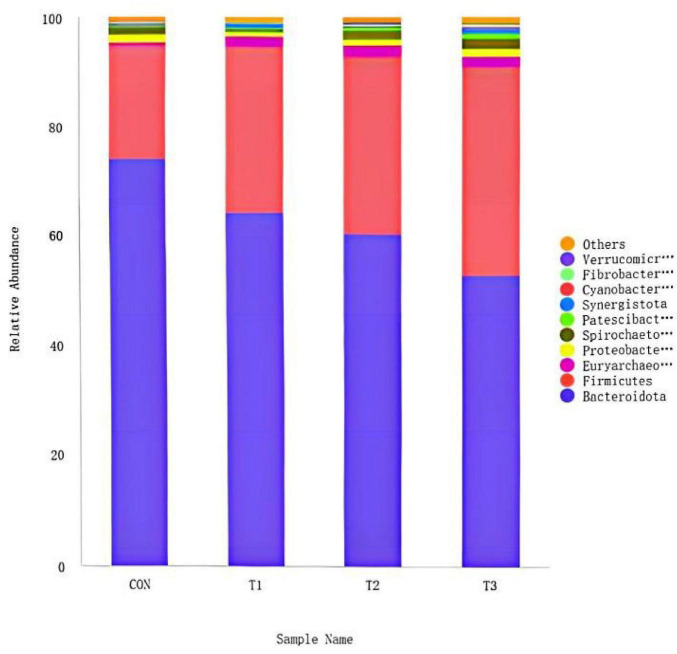
Distribution of main rumen bacteria at phylum level.

The ruminal microbial community structure at the phylum level for *Hu* lambs supplemented with varying doses of LE. The top 10 phyla by relative abundance were identified as *Bacteroidota*, *Firmicutes*, *Euryarchaeota*, *Proteobacteria*, *Spirochaetota*, *Patescibacteria*, *Synergistota*, *Cyanobacteria*, *Fibrobacterota*, and *Verrucomicrobiota*. *Bacteroidota* and *Firmicutes* dominated the microbial composition across all treatment groups. The CON group exhibited the highest relative abundance of *Bacteroidota* (74.06%), followed by T1 (64.36%), T2 (60.57%), and T3 (53.45%). In contrast, the relative abundance of *Firmicutes* was higher in all treatment groups compared to the CON group (21.04%), with values of 30.66% in T1, 32.74% in T2, and 37.88% in T3 (Figure 4).

#### 3.6.2 Effects of LE on the rumen microbial community structure (family level) in *hu* lambs

**FIGURE 5:**
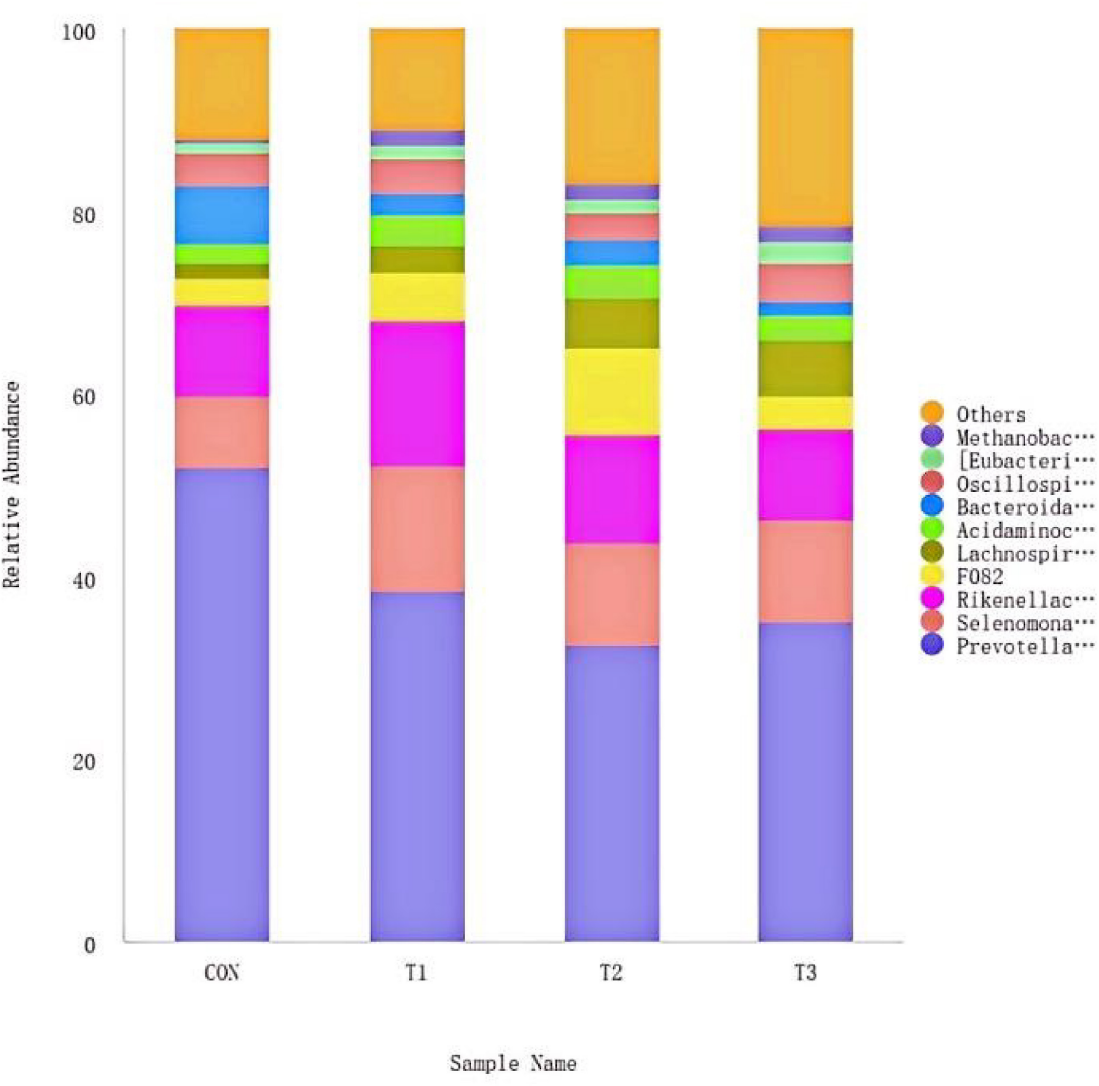
Distribution of main rumen bacteria at family level.

The effects of different levels of LE supplementation on the ruminal microbiota structure of *Hu* lambs at the family level. The top 10 most abundant families were *Prevotellaceae*, *Selenomonadaceae*, *Rikenellaceae*, *F082*, *Lachnospiraceae*, *Acidaminococcaceae*, *Bacteroidales_RF16_group*, *Oscillospiraceae*, *Eubacterium_coprostanoligenes_group*, and *Methanobrevibacteraceae*. Among these, *Prevotellaceae* was the dominant family in all groups. Its relative abundance was highest in the CON group (52.10%) compared to the treatment groups, with values of 38.49% in T1, 32.61% in T2, and 35.13% in T3. The relative abundance of *Prevotellaceae* was significantly lower in all treatment groups than in the CON group (*P* < 0.05). In contrast, *Lachnospiraceae* abundance was significantly higher in all treatment groups (*P* < 0.01), accounting for 2.80% (T1), 5.36% (T2), and 6.26% (T3), respectively. Furthermore, the T3 group exhibited a significantly higher abundance of *Lachnospiraceae* compared to the group T1, while the abundance of *Bacteroidales_RF16_group* in T3 was significantly lower than that in the CON group (Figure 5).

#### 3.6.3 The effect of LE on the rumen microbial community structure (genus level) in *hu* lambs

**FIGURE 6:**
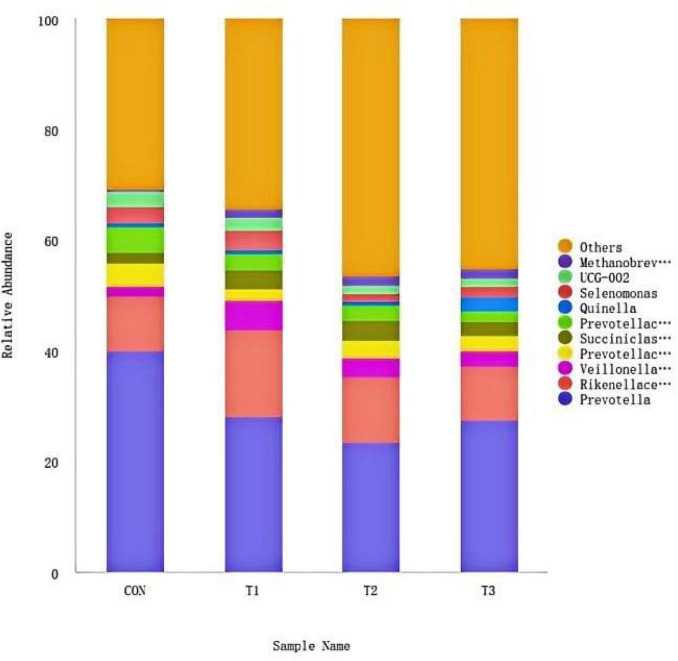
Distribution of main rumen bacteria at genus level.

The effects of varying levels of LE supplementation on the ruminal microbiota structure at the genus level in *Hu* lambs. The top 10 most abundant genera identified were *Prevotella*, *Rikenellaceae_RC9_gut_group*, *Veillonellaceae_UCG-001*, *Prevotellaceae_UCG-001*, *Succiniclasticum*, *Prevotellaceae_UCG-003*, *Quinella*, *Selenomonas*, *UCG-002*, and *Methanobrevibacter*. Among these, *Prevotella* and *Rikenellaceae_RC9_gut_group* were the dominant genera across all groups. The relative abundance of *Prevotella* was higher in the CON group (40.01%) compared to the treatment groups, with values of 28.10% in T1, 23.58% in T2, and 27.58% in T3. Although the relative abundance of *Prevotella* was lower in all treatment groups than in the CON group, the differences were not statistically significant (*P* > 0.05). The relative abundance of *Rikenellaceae_RC9_gut_group* was higher in T1 (15.74%) and T2 (11.71%) compared to the CON and T3 groups. No significant differences in genus-level relative abundances were observed among the treatment groups (Figure 6).

#### 3.6.4 LEfSe analysis

**FIGURE 7:**
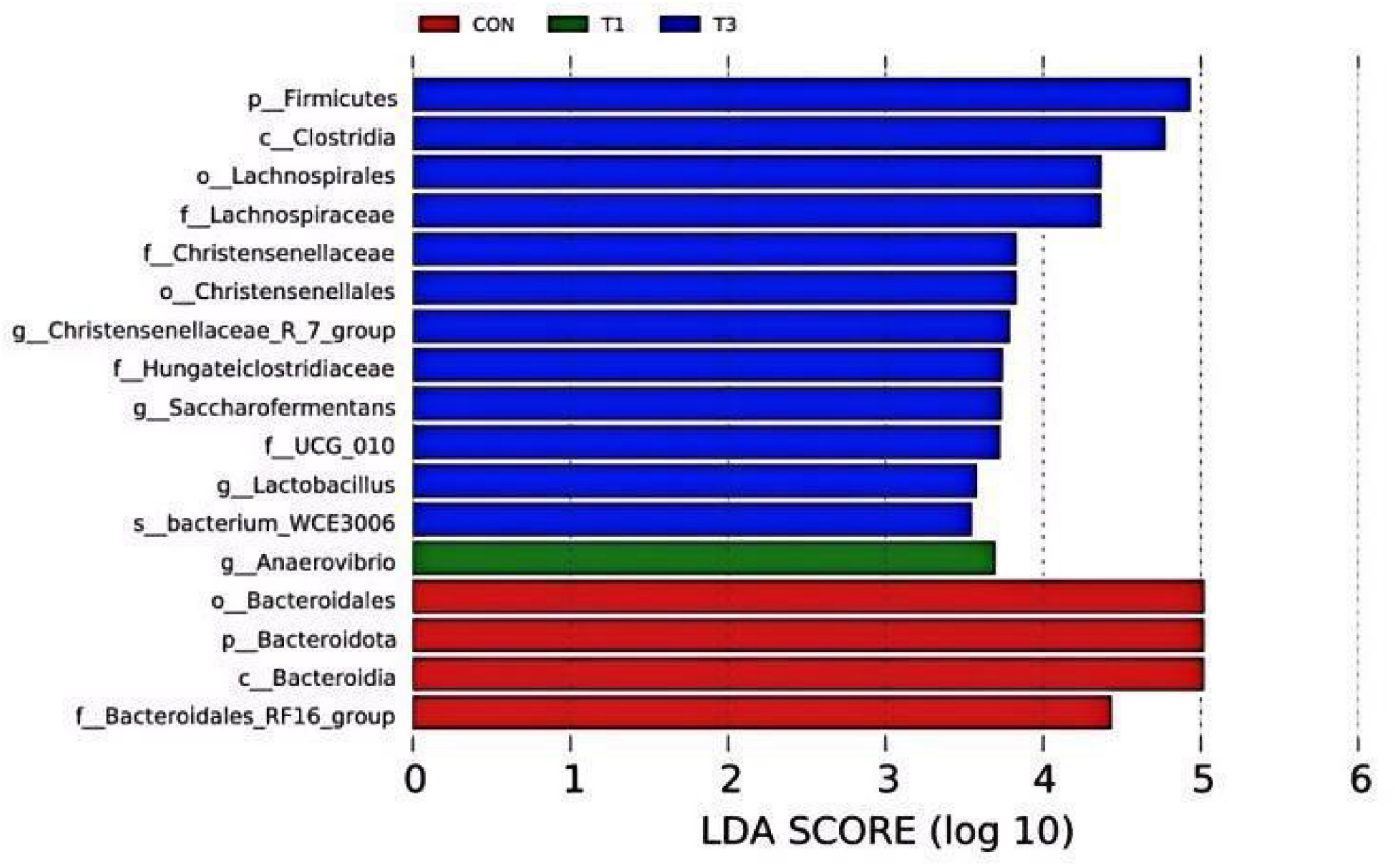
LDA Distribution.

LEfSe analysis was conducted to identify taxa with significant differences in relative abundance between the groups using Linear Discriminant Analysis (LDA). The histogram in Figure 7 shows taxa with an LDA score greater than 3.5, indicating significant differences in abundance. Seventeen microbial taxa in the rumen across the four treatment groups exceeded this threshold, including 2 phyla, 2 classes, 3 orders, 5 families, 4 genera, and 1 species. In the T3 group, the significantly enriched taxa included the phylum *Firmicutes*; the class *Clostridia*; the orders *Lachnospirales* and *Christensenellales*; the families *Lachnospiraceae*, *Christensenellaceae*, and *Hungateiclostridiaceae*; the genera *Christensenellaceae_R-7_group*, *Saccharofermentans*, *UCG-010*, *Lactobacillus*; and the species *bacterium WCE3006*. In contrast, the genus *Anaerovibrio* was significantly enriched in the T1 group. The CON group exhibited significant enrichment in the order *Bacteroidales*, phylum *Bacteroidota*, class *Bacteroidia*, and the family *Bacteroidales_RF16_group*.

#### 3.6.5 PICRUSt2 function prediction

**FIGURE 8:**
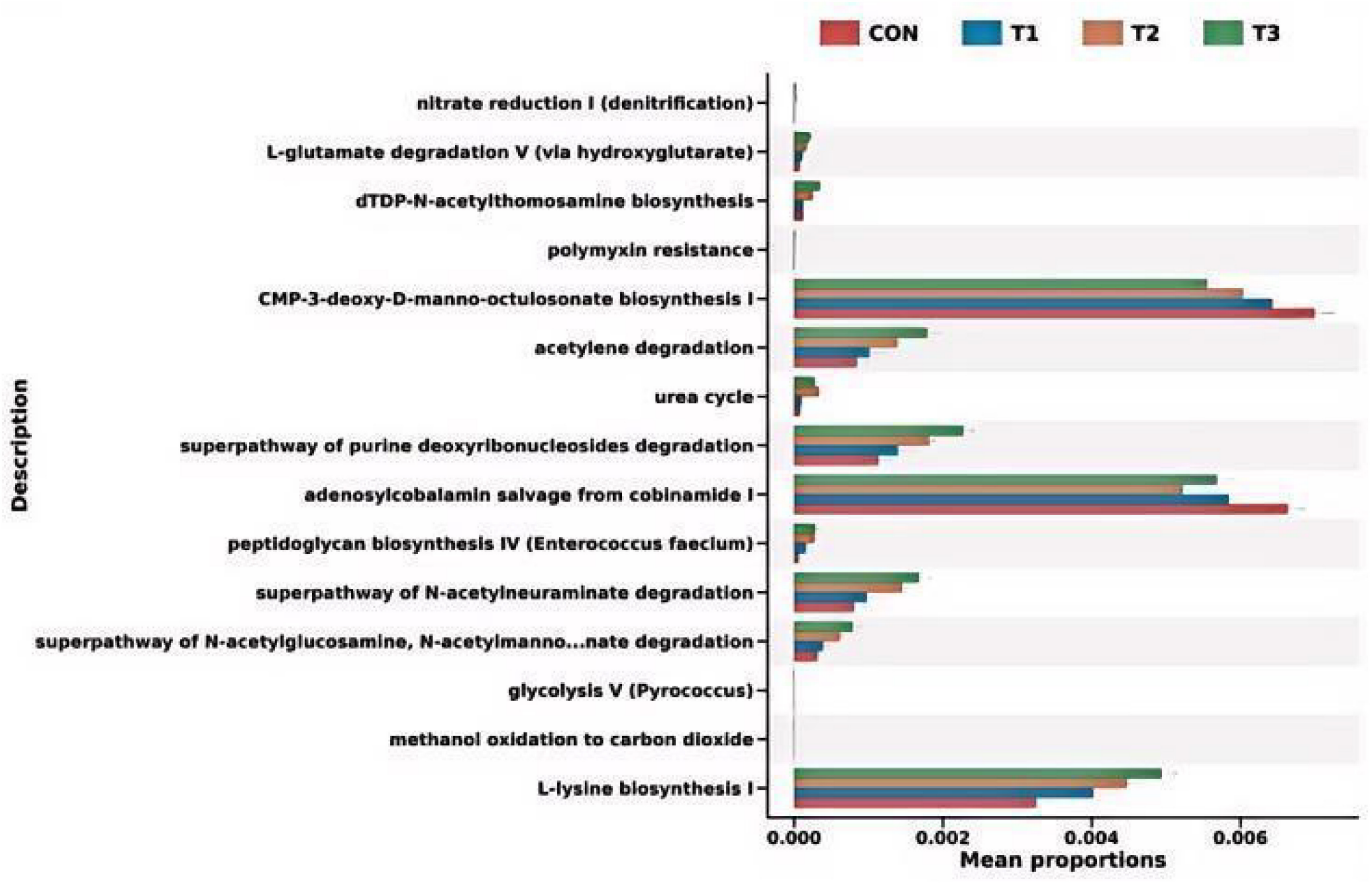
Differential metabolic pathways between groups.

The top 15 differentially ranked functions identified across the four groups, highlighting the enriched pathways. In the control group, two pathways were enriched: CMP-3-deoxy-D-manno-octulosonate biosynthesis I and adenosylcobalamin salvage from cobinamide I. In the T2 group, three pathways were enriched: nitrate reduction I (denitrification), urea cycle, and glycolysis V (Pyrococcus). Ten pathways were enriched in the T3 group: *L*-glutamate degradation V (via hydroxyglutarate), dTDP-N-acetylthomosamine biosynthesis, polymyxin resistance, acetylene degradation, superpathway of purine deoxyribonucleosides degradation, peptidoglycan biosynthesis IV (Enterococcus faecium), superpathway of N-acetylneuraminate degradation, superpathway of N-acetylglucosamine, N-acetylmannosamine, and N-acetylneuraminate degradation, methanol oxidation to carbon dioxide, and *L*-lysine biosynthesis I (Figure 8).

### 3.7 Effect of supplementing LE on the growth performance of *Hu* lambs

As shown in Table 4, there were no significant differences (*P* > 0.05) in Initial Body Weight (IBW), Final Body Weight (FBW), Average Daily Feed Intake(ADFI), Average Daily Gain (ADG) or Feed Conversion Ratio across all groups throughout the trial period.

**TABLE 4:** Effects of dietary LE supplementation on growth performance in *Hu* lambs (*n* = 7).

Items	Groups				*SEM*	*P*-value		
	CON	T1	T2	T3		Anova	Linear	Quadratic
Initial body weight (IBW) (kg)	20.31	20.20	20.15	20.19	0.10	0.96	0.68	0.73
Final body weight (FBW) (kg)	29.05	29.41	29.61	29.32	0.17	0.74	0.54	0.37
Average daily feed intake (ADFI) (kg)	1.44	1.52	1.51	1.46	0.11	0.43	0.25	0.29
Average daily gain (ADG) (g)	194.37	204.76	210.16	202.86	3.26	0.40	0.30	0.19
Feed conversion ratio (F:R)	7.40	7.43	7.17	7.18	0.14	0.89	0.58	0.77

Different lowercase letters on the same data point indicate significant differences (*P* < 0.05), different uppercase letters indicate highly significant differences (*P* < 0.01), and identical letters or no letters indicate no significant differences (*P* > 0.05).

### 3.8 Effect of supplementing LE on blood parameters in *Hu* lambs

As presented in Table 5, at 0 h before supplementation, T levels in the T2 and T3 groups were significantly higher than those in the CON group (*P* < 0.05), showing a linear increase. At 4 h after supplementation, no significant differences in T levels were observed among the groups (*P* > 0.05). Additionally, there were no significant differences in the levels of E_2_, GH, and INS among the groups at either 0 h before or 4 h after supplementation (*P* > 0.05).

**TABLE 5:** Effects of LE supplementation on plasma hormone levels in *Hu* lambs (*n* = 5).

Items	Time	Groups				*SEM*	*P*-value		
		CON	T1	T2	T3		Anova	Linear	Quadratic
Testosterone (T) (ng/mL)	0 h	0.62^b^	0.81^ab^	0.98^a^	0.94^a^	3.31	0.05	0.01	0.22
	4 h	1.03	1.17	1.22	1.24	0.82	0.50	0.17	0.54
Estradiol (E_2_) (pg/mL)	0 h	22.58	22.3	19.39	20.88	0.89	0.47	0.27	0.58
	4 h	21.02	20.17	17.72	19.94	0.47	0.71	0.54	0.46
Growth hormone (GH) (ng/mL)	0 h	4.83	5.08	5.06	5.22	0.10	0.96	0.63	0.94
	4 h	5.77	5.78	5.99	5.96	0.06	0.98	0.73	0.97
Insulin (INS) (uIU/mL)	0 h	14.95	14.04	13.91	12.79	0.59	0.63	0.22	0.93
	4 h	13.56	13.03	13.25	12.45	0.39	0.76	0.37	0.86

Different lowercase letters on the same data point indicate significant differences (*P* < 0.05), different uppercase letters indicate highly significant differences (*P* < 0.01), and identical letters or no letters indicate no significant differences (*P* > 0.05).

### 3.9 The effect of supplementing LE on plasma antioxidant indicators in *Hu* lambs

As shown in Table 6, the NO content in the experimental group showed a secondary change at 4 h post-supplementation (*P* < 0.05). However, no significant differences (*P* > 0.05) were found in the plasma activities of T-AOC, CAT, SOD, GSH-Px, or MDA content between the 0-h pre-supplementation and 4-h post-supplementation time points across all experimental groups.

**TABLE 6:** Effects of supplemental LE on plasma antioxidants in *Hu* lambs (*n* = 5).

Items	Time	Groups				*SEM*	*P*-value		
		CON	T1	T2	T3		Anova	Linear	Quadratic
Total antioxidant capacity (T-AOC) (U/mL)	0 h	74.60	73.21	73.71	80.38	0.67	0.58	0.34	0.34
	4 h	72.45	76.41	81.23	80.83	1.45	0.27	0.07	0.54
Catalase (CAT) (U/mL)	0 h	47.08	48.45	48.56	48.49	0.09	0.97	0.69	0.77
	4 h	52.33	51.02	52.45	55.75	1.17	0.35	0.18	0.24
Superoxide dismutase (SOD) (U/mL)	0 h	4.48	4.50	3.50	3.82	2.39	0.11	0.06	0.64
	4 h	3.49	3.47	3.55	3.42	0.05	0.99	0.93	0.82
Glutathione peroxidase (GSH-Px) (U/mL)	0 h	9.95	9.48	9.88	9.65	1.48	0.26	0.53	0.52
	4 h	10.36	10.32	10.28	10.37	0.05	0.98	0.96	0.73
Malondialdehyde (MDA) (nmol/mL)	0 h	154.82	152.82	159.93	155.03	0.18	0.91	0.80	0.84
	4 h	170.92	161.62	161.78	170.47	0.67	0.58	0.97	0.18
Nitric oxide (NO) (μmol/L)	0 h	49.85	52.13	51.39	45.42	1.80	0.19	0.18	0.08
	4 h	57.64^a^	49.37^b^	49.84^b^	52.67^ab^	4.62	0.02	0.09	<0.01

Different lowercase letters on the same data point indicate significant differences (*P* < 0.05), different uppercase letters indicate highly significant differences (*P* < 0.01), and identical letters or no letters indicate no significant differences (*P* > 0.05).

### 3.10 The effect of LE on plasma protein levels in *Hu* lambs

As shown in Table 7, no significant differences were observed in the levels of TP, ALB, and GLB at 0 h before or 4 h after supplementation among the experimental groups compared to the CON group (*P* > 0.05).

**TABLE 7:** Effects of LE on plasma proteins in *Hu* lambs (*n* = 5).

Items	Time	Groups				*SEM*	*P*-value		
		CON	T1	T2	T3		Anova	Linear	Quadratic
Total protein (TP) (g/L)	0 h	61.78	60.24	61.66	60.92	0.23	0.88	0.77	0.79
	4 h	57.70	56.96	57.20	57.22	0.08	0.97	0.81	0.73
Albumin (ALB)(g/L)	0 h	31.56	32.52	32.76	32.26	0.21	0.89	0.65	0.53
	4 h	31.56	30.20	31.16	30.42	0.32	0.81	0.63	0.79
Globulin (GLB)(g/L)	0 h	30.22	27.92	28.90	28.66	0.18	0.91	0.72	0.66
	4 h	26.14	26.76	26.04	26.80	0.06	0.98	0.87	0.97

Different lowercase letters on the same data point indicate significant differences (*P* < 0.05), different uppercase letters indicate highly significant differences (*P* < 0.01), and identical letters or no letters indicate no significant differences (*P* > 0.05).

### 3.11 Correlation analysis between growth performance of *Hu* lambs and rumen microorganisms

**FIGURE 9:**
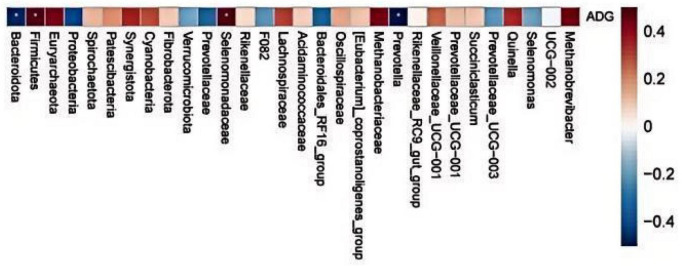
Relationship between ruminal microbiota features and lamb growth performance traits.

The results of correlation analysis between the growth performance of *Hu* lambs and the rumen microbiota in Figure 9. At the phylum level, the abundance of *Firmicutes* in rumen fluid showed a significant positive correlation with the ADG of *Hu* lambs (*P* < 0.05), while the abundance of *Bacteroidota* in rumen fluid exhibited a significant negative correlation with ADG (*P* < 0.05). At the family level, the abundance of *Selenomonadaceae* in rumen fluid demonstrated a significant positive correlation with ADG (*P* < 0.05). At the genus level, *Prevotella* abundance in rumen fluid was negatively correlated with ADG (*P* < 0.05).

### 3.12 Phylum level correlation analysis

**FIGURE 10:**
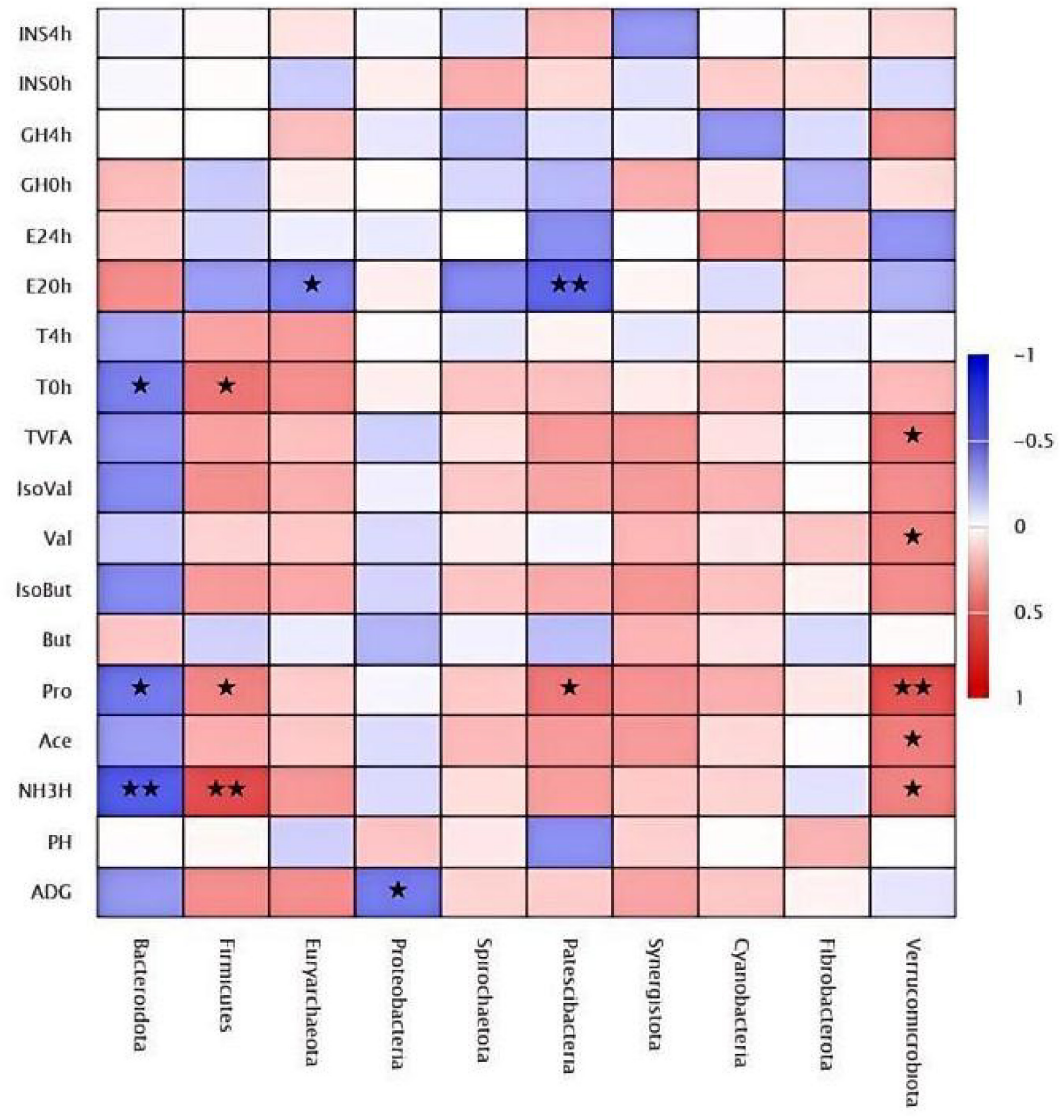
Clustered heatmap of phylum-level correlation analysis. *Indicates *P* < 0.05. **Indicates *P* < 0.01.

The results of a correlation analysis investigating the relationships between LE supplementation and growth performance, reproductive hormone indicators, and rumen microbiota in *Hu* lambs, which showed in Figure 10. At the phylum level, the top 10 bacterial phyla correlated with growth performance and hormone levels were *Bacteroidota*, *Firmicutes*, *Euryarchaeota*, *Proteobacteria*, *Spirochaetota*, *Patescibacteria*, *Synergistota*, *Cyanobacteria*, *Fibrobacterota*, and *Verrucomicrobiota*. Among these, *Patescibacteria* was significantly negatively correlated with ADG (*P* < 0.05), while it was positively correlated with rumen fermentation parameters (*P* < 0.05). Additionally, *Firmicutes* and *Verrucomicrobiota* exhibited a highly significant positive correlation with fermentation parameters (*P <* 0.01). Regarding hormone levels, *Bacteroidota* and *Euryarchaeota* were significantly negatively correlated (*P* < 0.05), and *Patescibacteria* showed a highly significant negative correlation with hormone levels (*P* < 0.01).

### 3.13 Correlation analysis of family levels

**FIGURE 11:**
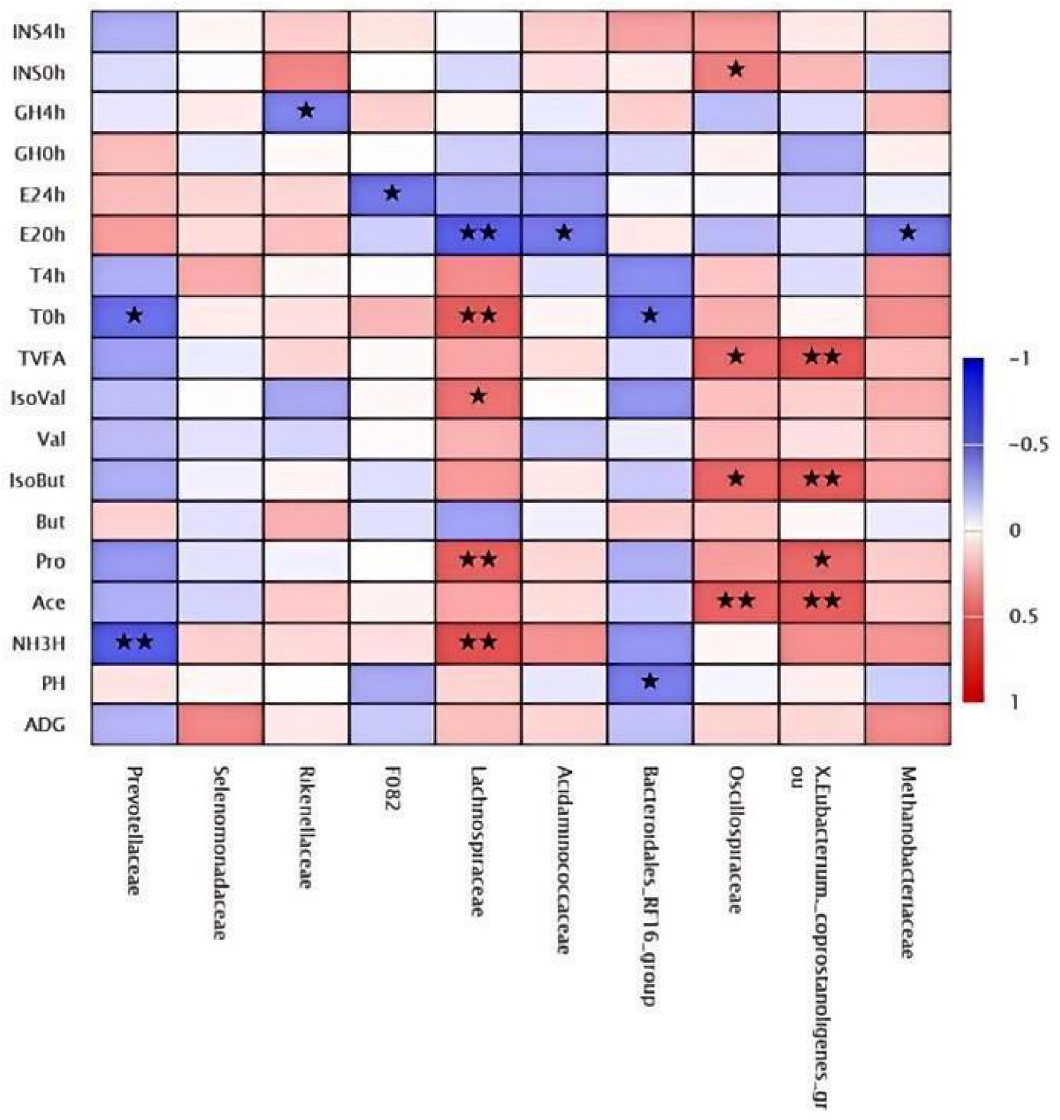
Clustered heatmap of family-level correlation analysis. *Indicates *P* < 0.05. **Indicates *P* < 0.01.

The top 10 bacterial families correlated with growth performance, hormone levels, and rumen fermentation parameters illustrated Figure 11, which includes *Prevotellaceae*, *Selenomonadaceae*, *Rikenellaceae, F082*, *Lachnospiraceae*, *Acidaminococcaceae*, *Bacteroidales_RF16_group*, *Oscillospiraceae*, *Eubacterium_coprostanoligenes_group*, and *Methanobrevibacteraceae*. At the family level, no significant correlation with ADG was observed, although a general positive trend was observed. Regarding rumen fermentation parameters, *Lachnospiraceae*, *Oscillospiraceae*, and *Eubacterium_coprostanoligenes_group* exhibited a highly significant positive correlation (*P* < 0.01). In terms of hormonal associations, a significant negative correlation was identified between estrogen levels at 4 h and the family *F082* (*P* < 0.05), while *Rikenellaceae* showed a significant negative correlation with GH levels at 4 h (*P* < 0.05).

### 3.14 Correlation analysis of genus levels

**FIGURE 12:**
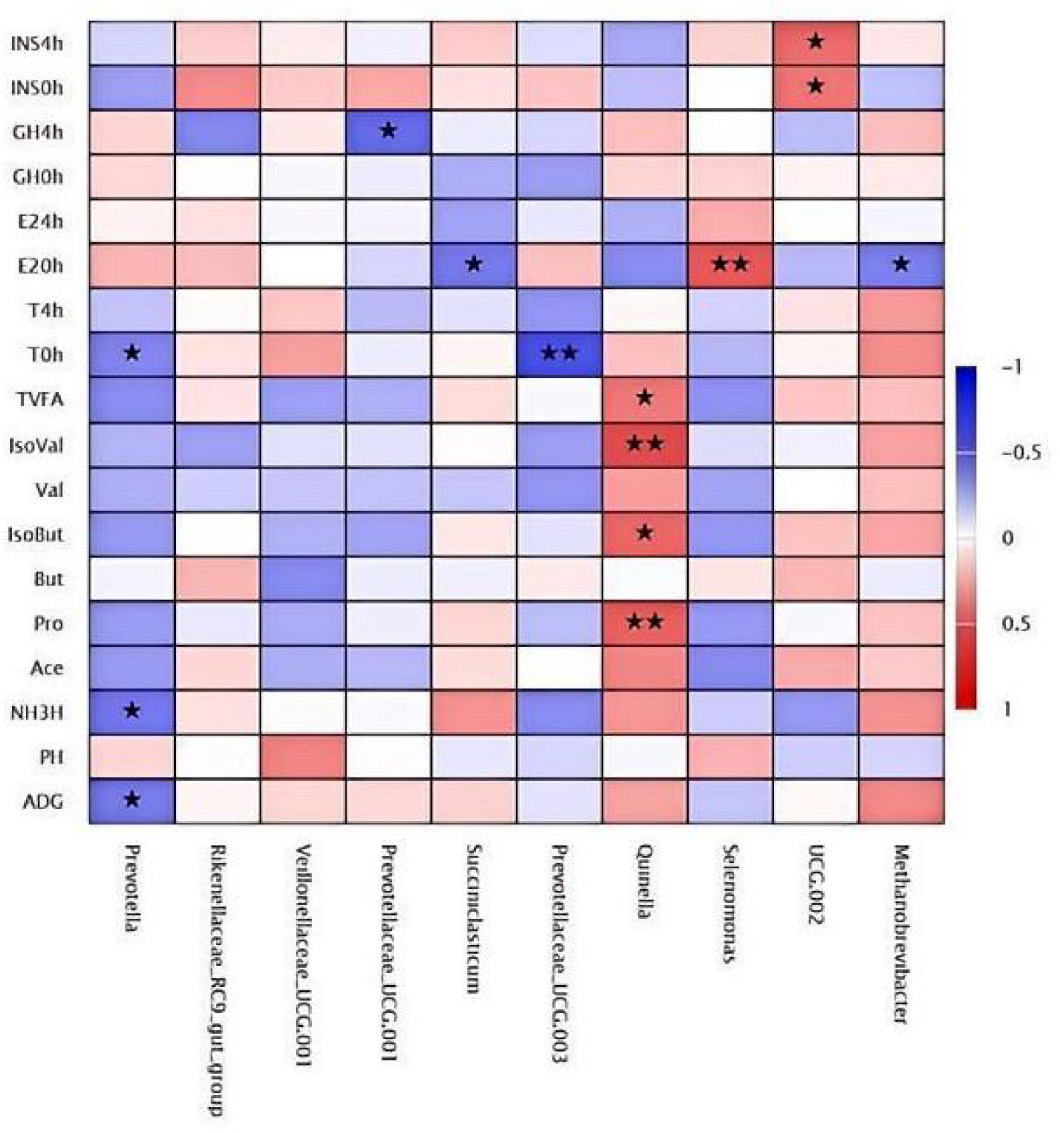
Clustered heatmap of genus-level correlation analysis. *Indicates *P* < 0.05. **Indicates *P* < 0.01.

The effects of varying levels of LE supplementation on the rumen microbiota structure at the genus level in *Hu* lambs, which presents Figure 12. The top 10 most abundant genera were *Prevotella*, *Rikenellaceae_RC9_gut_group*, *Veillonellaceae_UCG-001*, *Prevotellaceae_UCG-001*, *Succiniclasticum*, *Prevotellaceae_UCG-003*, *Quinella*, *Selenomonas*, *UCG-002*, and *Methanobrevibacter*. At the genus level, *Quinella* showed a highly significant positive correlation with propionate and isovalerate (*P* < 0.01) and a significant positive correlation with isobutyrate and total volatile fatty acids (TVFA) (*P* < 0.05). The genus *UCG-002* exhibited a significant positive correlation with INS levels (*P* < 0.05). In contrast, the genera *Prevotellaceae_UCG-003*, *Succiniclasticum*, *Prevotella*, *Prevotellaceae_UCG-001*, and *Methanobrevibacter* were negatively correlated with hormone levels. Notably, *Prevotellaceae_UCG-003* showed a highly significant negative correlation with T concentration at 0 h (*P* < 0.01).

## 4 Discussion

The efficient degradation of feed in ruminants largely depends on the homeostasis of the ruminal internal environment, which is closely associated with rumen pH, NH_3_-N concentration, and VFA levels (Castro-Montoya et al., 2011). Rumen pH serves as a critical physiological indicator of this balance, and deviations from the normal range (typically 5.5–7.5) can disrupt fermentation processes (Qiu et al., 2022). In the present study, rumen pH values across all lamb groups remained within the normal physiological range following LE supplementation, indicating that LE administration did not significantly affect ruminal fermentation.

The NH_3_-N in the rumen is derived from the microbial breakdown of non-fiber feed components. It serves as a key nitrogen substrate in microbial anabolism, supporting the growth and proliferation of bacteria and other microorganisms essential for microbial protein synthesis (Jefferson et al., 2022). Receptors for steroid sex hormones, such as T, E_2_, and progesterone (P), have been identified in the cytoplasm and nuclei of rumen bacteria. The binding of these hormones to their receptors can influence ruminal metabolism in ruminants (Han, 2006). Chen et al. (1999) demonstrated that daidzein supplementation via duodenal infusion in male water buffaloes increased blood T levels and significantly elevated ruminal concentrations of both NH_3_-N and TVFA. The increase in ruminal NH_3_-N concentration following LE-induced elevation in plasma T is likely mediated by T binding to microbial hormone receptors. This suggests that elevated T may interact with hormone receptors on rumen microbes, modulating the microbial community and thereby enhancing NH_3_-N production.

Short-chain fatty acids (SCFAs) in the rumen are primarily synthesized through microbial fermentation of feed. The VFAs, a major component of SCFAs, serve as the primary energy source for ruminants, providing 70-80% of their total energy needs (Kumar et al., 2024). The present study demonstrated that LE supplementation induced a significant shift in the ruminal A:P, indicating a broader modulatory effect of LE on rumen fermentation. Propionate is a critical contributor to weight gain in ruminants, with its adequate availability being essential for maintaining healthy growth (Zhang et al., 2022). A highly significant linear increase in ruminal propionate concentration was observed with increasing doses of LE, likely due to T-enhanced microbial carbohydrate degradation. However, this shift in ruminal fermentation did not result in improved growth performance, such as ADG, in lambs. This lack of response may be attributed to the relatively short trial duration, suggesting that further investigation is needed to determine the optimal dosage and duration of LE supplementation for maximizing growth performance.

The rumen of ruminants harbors a complex consortium of symbiotic microorganisms, including bacteria, archaea, ciliates, fungi, and viruses (Firkins and Yu, 2015). Among these, the phyla *Firmicutes* and *Bacteroidetes* are the most abundant, containing numerous fibrolytic genera such as *Succiniclasticum*, *Prevotella*, and *Ruminococcus*, which form the core ruminal microbiota (Mizrahi et al., 2021). These microbes play a critical role in degrading plant fibers and polysaccharides, fermenting them into intermediates such as succinate, lactate, and fumarate, which are then converted into end products including VFAs, microbial protein, and other nutrients that provide energy to the host (Schwab and Broderick, 2017). Consequently, the rumen microbiota is central to the digestive and metabolic processes of ruminants. A key finding of this study was the significant alteration in the core ruminal microbiota following LE supplementation, specifically a decrease in the relative abundance of *Bacteroidetes* and an increase in *Firmicutes* compared to the CON group. Furthermore, the correlation analysis between the growth performance of *Hu* sheep lambs and rumen microbiota revealed a significant positive correlation between the relative abundance of Firmicutes and the average daily gain (ADG) of lambs. Given that *Firmicutes* are involved in the conversion of dietary fiber to VFAs (Ko et al., 2020), while *Bacteroidetes* are critical for polysaccharide breakdown, this shift suggests a potential modification in the ruminal metabolic landscape. Previous studies have linked a higher *Firmicutes-to-Bacteroidetes* (F/B) ratio with improved growth performance (Bauman et al., 2011; Huangfu, 2023). Our correlation analysis between the rumen microbiota and growth parameters in lambs produced similar findings, further supporting this relationship. The observed increase in TVFA and propionate levels may be attributed to the elevated relative abundance of *Firmicutes*. At the genus level, *Succiniclasticum* abundance was consistently higher in all treatment groups compared to the CON group. This genus is known for its ability to degrade fiber to produce succinate, which is then converted to propionate, providing energy to the host. Therefore, the increase in *Succiniclasticum* abundance likely contributed to the elevated propionate concentration observed in this study.

The growth, development, and metabolic processes of animals are regulated by various factors, with hormones and growth factors playing pivotal roles (Mei, 2024). In male animals, T, as a primary reproductive hormone, not only exerts hormonal effects but also regulates critical physiological processes such as appetite control, protein metabolism, and lipid metabolism (Yutong et al., 2025). During osteoblast differentiation, T helps maintain bone growth and ameliorate osteoporosis (Vanderschueren et al., 2014). Endogenous T has also been found to suppress the response of melanin-concentrating hormone (MCH) neurons to glucose, thereby reducing post-fasting plasma glucose levels, enhancing induced satiety, and increasing feed intake (Fukushima et al., 2015). It has further been shown to promote protein synthesis in skeletal muscle and facilitate skeletal growth, contributing to improved overall growth performance in animals (McClure et al., 2000; Zhang et al., 2019). Research indicates that sustained T circulation in lambs has both direct and indirect effects on muscle protein synthesis and bone growth, which collectively enhance growth performance (Schanbacher et al., 1980). A study by Li et al. (2019) found that laying hens supplemented with 0.5 mg/d of LE for 6 weeks showed a significant increase in body weight. Similarly, Rezaei et al. (2020) demonstrated that LE supplementation in goats significantly increased ADG and F:G, positively affecting growth performance. However, in the present study, no significant changes in growth performance were observed in lambs following LE supplementation. Existing research indicates that T plays a role in regulating both protein and lipid metabolism, promoting protein anabolism while also stimulating lipid catabolism (Guo, 2015). Lipid metabolism is particularly crucial for the short-term improvement of animal growth performance. In this trial, although LE supplementation increased circulating T concentrations in lambs, it did not result in enhanced ADG. It is hypothesized that the weight gain from T-stimulated muscle protein synthesis may have been counteracted by concurrent lipid catabolism, leading to no net improvement in overall growth performance.

Animal growth is regulated by GH, and elevated T levels can stimulate GH secretion, which, in turn, plays a key role in various developmental and metabolic processes such as protein synthesis, growth promotion, and lipolysis (Song et al., 2023). In contrast, INS exerts opposing effects to GH in lipogenesis, enhancing tissue glucose uptake and fatty acid synthesis by promoting fatty acid synthase (FAS) expression (Guo, 2015). As a highly selective cytochrome P450 aromatase inhibitor, LE effectively blocks the conversion of T to E_2_, thereby elevating systemic T levels. This action is hormonally selective and does not interfere with the normal secretion of corticosteroids or thyroid hormones (Liu et al., 2024). Fu (2023) demonstrated that oral administration of varying doses of LE (0.1, 1.0, and 10 mg/kg/d) to female juvenile mice reduced ovarian E_2_ levels and increased T levels. Similarly, Ortiz-Carrera et al. (2019) observed a significant increase in serum T levels following supplementation with 2.5 mg/d LE in male goats, with serum E_2_ levels remaining unchanged. These findings align with the results of the present study.

Plasma biomarkers such as TP, ALB, and GLB—which reflect protein assimilation, transport and repair, and immune function, respectively (Peng et al., 2020)—were analyzed. All concentrations were within normal physiological ranges, and no significant differences were observed among groups. This suggests that LE supplementation did not have an overt impact on these parameters under the current experimental conditions. Therefore, the potential immunomodulatory effects of LE in *Hu* lambs warrant further investigation under different conditions or with more targeted immune challenges.

During metabolic processes, organisms generate various highly reactive oxygen species (ROS), reactive nitrogen species (RNS), and singlet oxygen. When present in excessive concentrations, these molecules can damage intracellular macromolecules, such as DNA and proteins, contributing to the pathogenesis of numerous diseases (Pisoschi and Pop, 2015). It has been suggested that androgens help mitigate oxidative stress in organisms. A study by Wang (2023) found that T deficiency exacerbated oxidative damage in the hippocampal region of male APP/PS1 mice, a model of Alzheimer’s disease. Additionally, Yao (2022) reported that during heat stress, castrated beef cattle showed significantly lower activities of T-AOC, SOD, and GSH-Px compared to partially castrated and sham-operated groups. Chen (2016) observed that LE supplementation in bulls increased T-AOC and reduced MDA content in seminal plasma. As an aromatase inhibitor, LE works by inhibiting aromatase activity, thus blocking the conversion of androstenedione (AD) and T to estrogens, which indirectly elevates androgen levels in muscle tissue (Bai et al., 2015). In the present study, LE supplementation did not significantly affect most plasma antioxidant parameters (T-AOC, CAT, SOD, GSH-Px) or MDA levels in *Hu* lambs. However, a notable decrease in NO concentration was recorded 4 h after supplementation in the T1 and T2 groups. The discrepancy between these results and previous findings may be attributed to the varying stress conditions inherent to the experimental models.

**FIGURE 13:**
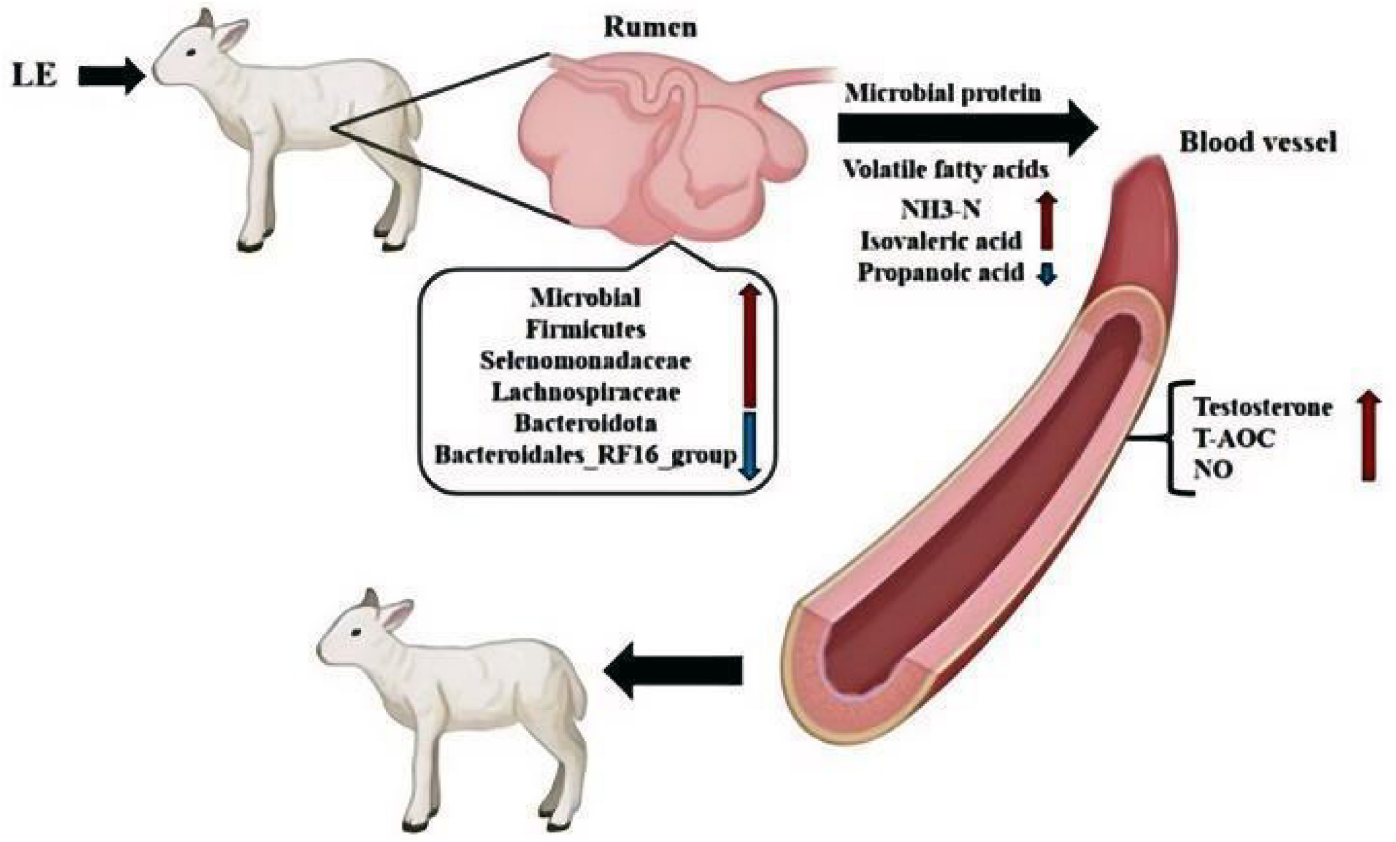
Effect of LE on rumen microflora structure and blood biochemical indexes of lambs.

In summary, dietary LE supplementation appears to enhance the ruminal fermentation pattern and fiber degradation capacity by modulating the microbial community (Figure 13). This aligns with previous research identifying E_2_, P, and T in ruminant rumen fluid, with T shown to promote ruminal fermentation (Han, 2006). Additionally, Silva et al. (2024) reported greater ruminal microbial diversity in male ruminants compared to females. Alpha-diversity indices (including Observed OTUs, Chao1, and Simpson) reflect community richness and diversity. In this study, Group T3 exhibited a significantly higher number of OTUs compared to the CON group, with an even more pronounced increase compared to Group T1. Furthermore, all alpha-diversity indices (OTUs, Chao1, Simpson) showed significant enhancement with increasing LE doses. These findings collectively suggest that LE supplementation increased both the diversity and richness of the ruminal microbiota in lambs, likely due to LE’s inhibition of androgen aromatization, which indirectly shaped the ruminal microbial environment.

## 5 Conclusion

Under the experimental conditions, LE supplementation increased lambs’ concentrations of NH_3_-N, propionic acid, and isovaleric acid, elevated the relative abundance of the *Firmicutes*, reduced the relative abundance of the *Bacteroidetes*, altered rumen fermentation patterns, and promoted an increase in plasma testosterone concentration. In comparison, LE supplementation at a dose of 0.2 mg/kg BW yielded superior effects.

## Data Availability

The data presented in this study are publicly available. The data can be found here: https://www.ncbi.nlm.nih.gov/sra, accession PRJNA1353064.
